# 24-month efficacy of bioactive versus fluoride sealant for non-cavitated occlusal caries in adults: a randomized clinical trial

**DOI:** 10.1038/s41598-025-29730-8

**Published:** 2025-12-11

**Authors:** Alaa Fathi Abdelsalam, Omar Shaalan

**Affiliations:** Conservative Dentistry Department, Faculty of Dentistry, Cairo University, Cairo, Egypt

**Keywords:** AAPD, Adults, Bioactive, Caries, Inhibition, Retention, Sealant and SmartCap, Diseases, Health care, Medical research

## Abstract

There is a recent suggestion by the American Academy of Pediatric Dentistry to seal initial non-cavitated carious lesions in adults. The aim of the present study was to evaluate the sealant retention and inhibition of initial carious lesion progression of the bioactive fissure sealant with SmartCap technology “Biocoat” compared to fluoride releasing fissure sealant “Clinpro sealant” over 24 months in non-cavitated occlusal carious lesions in adults. The present study is a randomized clinical trial, following a parallel group design with superiority framework and 1:1 allocation ratio. Thirty-six participants with 64 non-cavitated occlusal carious lesions were divided into two groups according to random allocation sequence (n = 32); either Biocoat (intervention) or Clinpro (control). Sealants were assessed at baseline, 12 and 24 months for retention and caries progression. Statistical analysis was performed by MedCalc 22 software for Windows. Intergroup comparison between sealants within each follow-up period was done using the Chi-Square test (*p* ≤ 0.05). Intragroup comparison within each intervention to assess change in clinical performance through time was performed by the Cochran’s Q test (*p* ≤ 0.016). After 24 months, two sealants in Biocoat group and 11 sealants in Clinpro group failed due to loss of retention or caries progression. Biocoat has shown 93.75% success rate, while Clinpro has shown 65.62% success rate after 24 months. The relative risk (RR) was 0.18 (95% CI 0.04374 to 0.7558), showing 82% less risk of failure of Biocoat when compared to Clinpro sealant, and this was statistically significant (*p* = 0.0190). Biocoat sealant showed better retention rate and inhibition of initial carious lesion progression after 24 months when compared to fluoride releasing sealant in non-cavitated occlusal caries in adults.

*Clinical relevance*: The present study supports the suggestion of AAPD on sealing initial carious lesions. Sealing of initial non-cavitated occlusal carious lesions is a conservative and successful approach and should be implemented in routine dental practice in adults with recent history of dental caries.

*Trial registration*: The present clinical trial was retrospectively registered on clinicaltrials.gov on 22-05-2023 (NCT05891288).

## Introduction

Dental caries is among the most prevalent diseases affecting people globally^1^. In Egypt, dental caries is estimated to affect approximately 60% of the population^2^. Preventing dental caries requires the early detection of initial carious lesions and early assessment of the individual’s caries risk. Prevention of dental caries can by categorized into: primary, secondary and tertiary levels of prevention^3^. Among the possible treatment options within the secondary level of prevention is sealing of retentive pits and fissures as soon as the molar erupts. However, sealing of permanent molars in young adults has been neglected^4^.

A 2016 guideline panel, formed by the American Dental Association Council on Scientific Affairs (ADA CSA) and the American Academy of Pediatric Dentistry (AAPD), established evidence-based clinical guidelines for the application of pit-and-fissure sealants on the occlusal surfaces of primary and permanent molars. This was based on a systematic review which concluded that sealants applied to the occlusal surfaces of primary and permanent molars are effective in preventing carious lesions and arresting non-cavitated carious lesions, in comparison to no treatment or the application of fluoride varnish^5^. Since no studies have been found on sealants’ effects on caries prevention and arrest of non-cavitated carious lesions in occlusal surface of adults, the guideline panel advises clinicians and patients to expect similar treatment effects in other age groups, particularly in adults with a recent history of dental caries^4^.

Moreover, the Japanese Society of Conservative Dentistry (JSCD) recommended to seal incipient pit and fissure enamel caries in adults^6^. Posterior teeth may remain vulnerable for caries initiation and progression many years after eruption, thus sealing non cavitated carious pits and fissures may be beneficial in young adults to prevent or arrest initial non cavitated carious lesions in enamel^7,8^. Present evidence suggests that sealants are excellent treatment option to prevent the progression of incipient lesions, including ICDAS 1, 2, and 3 lesions and ICDAS 4 lesions in the outer third of dentin (radiographically determined), while ICDAS 5 and 6 require restorative treatment^9^.

The success rate of fissure sealants for prevention of dental caries is about 61% after an evaluation period of 5 years^10^. Various materials have been postulated to be used for sealing pits and fissure. Mainly they can be classified into 3 main categories: Resin-based, glass ionomer and hybrid fissure sealants including compomers and giomers^8^. Prevention potential for both resin-based and glass ionomer sealants was 92% after 6 months^11^. After 18 months resin-based sealants showed prevention capability ranging from 64 —88%, while glass ionomer sealants showed caries prevention potential in 88% of cases^12^. After 5 years resin-based and glass ionomer fissure sealants showed prevention potential of 61% and 35% respectively^10^. Compomers, giomers and bioactive fissure sealants had limited evidence based information in the literature^8^. Resin-based fissure sealants showed retention rate and wear resistance higher than glass ionomer, however glass ionomer showed similar or higher caries prevention potential within 2 years. This may be attributed to the antibacterial activity and the remineralization potential of fluoride releasing glass ionomer. Previous research^13,14^ concluded that the clinical evidence currently available indicates that the complete retention of pit and fissure sealants may not be a valid surrogate endpoint for caries prevention and that additional research is required to confirm the existing findings. As an example for that, in partially or completely lost glass ionomer sealant, remnants of the ion-enriched layer created due to chemical interaction between polyacrylic acid and hydroxyapatite remains inside pits and fissures even after complete loss of glass ionomer sealants, continuing the caries preventive effect^15^.

**Fig. 1:**
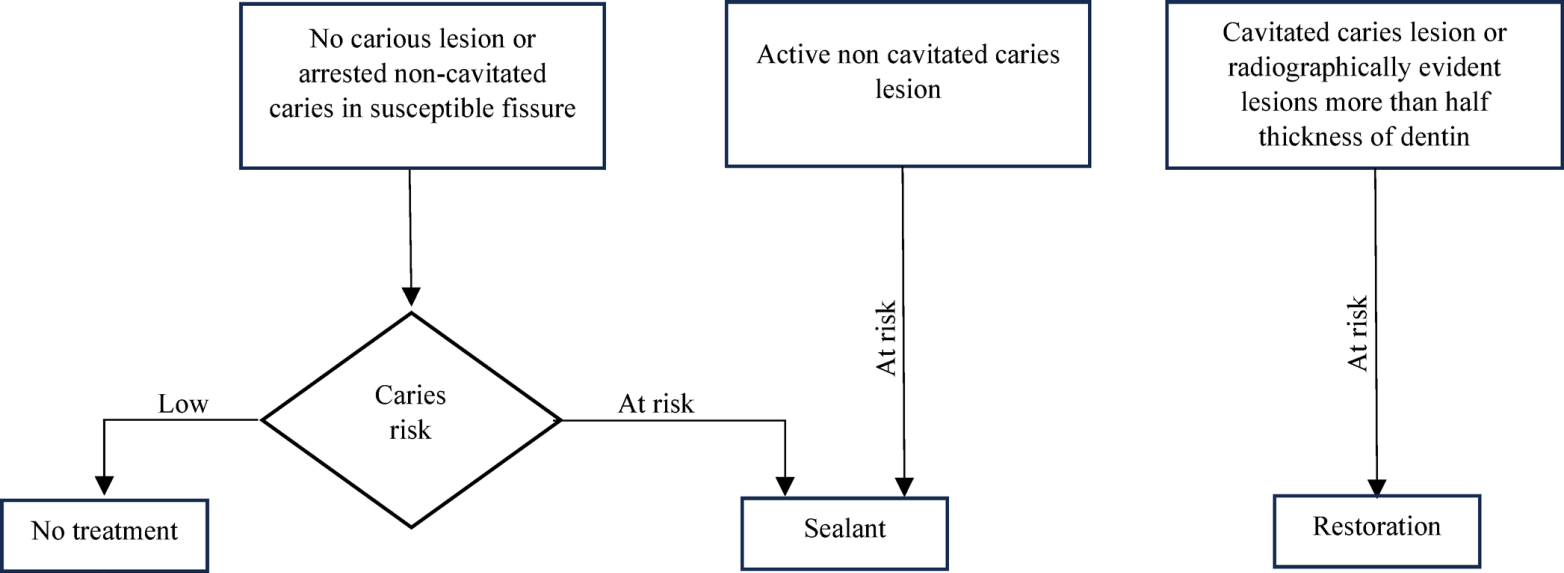
Current recommendations for using pits and fissure sealants in permanent teeth^16^.

As previously mentioned, there is strong evidence for sealing initial carious lesions, yet sealant utilization in such cases is not adopted by dentists^4^. One of the main obstacles is the fear of sealing over active carious lesions, which is difficult to assess after sealant application. The current recommendations for using pits and fissure sealants in permanent teeth is shown in Fig. 1^16^.

There was a need to develop a sealant material combining the merits of resin-based sealants and glass ionomer sealants, in other words combining retention and wear resistance with remineralization and caries inhibition. A bioactive resin based pits and fissures sealant was developed by Premier ® utilizing the SmartCap technology, where rechargeable semi-porous ion-enriched resinous microcapsules were incorporated in the resin matrix of the sealant, thus enhancing sealant retention and caries inhibition potential^7^.

There is limited evidence-based information and knowledge gap in the literature regarding sealing initial carious lesions in permanent molars of adults^4^. Therefore, the aim of the current study was to assess the retention rate and inhibition of initial carious lesion progression of the newly introduced bioactive fissure sealant with SmartCap technology compared to fluoride releasing fissure sealant after 24 months. The null hypothesis tested that there will be no difference in retention rate and inhibition of initial carious lesion progression between both sealants.

## Methods

### Trial registration, ethical approval and study design

The present clinical trial was retrospectively registered on clinicaltrials.gov on 22-05-2023 (NCT05891288), this was due to an administrative oversight. However, the final study protocol and outcomes were established and locked before patient enrollment and were not altered. The study was reported following the Consolidated Standards of Reporting Trials (CONSORT) guidelines created in 2010. Procedures of the present study involving human participants were abiding to the Helsinki declaration in 2013 and was approved by the research ethics committee at Faculty of Dentistry, Cairo University (10–22). The present randomized clinical trial followed a parallel group design with 1:1 allocation ratio and superiority framework.

### Sample size calculation

In a previous study^17^ , 39 out of 60 pit and fissure sealants in non-cavitated occlusal caries were functional after two to three years with a rate of 65%. In order to detect a difference of 30%, the calculated sample size was 27 teeth per group. This was increased by 20% to compensate for possible dropouts to reach 32 teeth in each group. Sample size calculation was performed using G*Power version 3.1.9.2 for windows (Heinrich Heine, Universität Düsseldorf, Düsseldorf, Germany)^18^ using Z test for difference between two independent proportions, using type I error of 5% and power of 80%.

### Eligibility criteria

The inclusion criteria of participants in the current study were: high caries risk according to caries management by risk assessment (CAMBRA) guidelines^19–21^, good oral hygiene and age range between 19 to 25 years. Molars with initial non-cavitated carious lesions with ICDAS II score 1 or 2, VistaProof light fluorescent camera reading more than 0.9 and less than 1.5 indicating early stages of enamel caries as per manufacturer recommendation^22^, normal contact with opposing dentition, no previous restorative treatment in other tooth surfaces were included. Patients with poor oral hygiene, disabilities, systemic diseases, rampant caries, xerostomia, lack of compliance, periodontal disease, evidence of bruxism and temporomandibular joint disorders were excluded. Molars with sound fissures, cavitated carious lesions, severe periodontal affection, signs of pulpal pathology and hypersensitivity were excluded.

### Recruitment and study settings

Participants in the present trial were recruited from the conservative dentistry department, Faculty of Dentistry at Cairo University, using non-probability convenient sampling. Eligible participants were enrolled, and all procedures were explained in detail, written informed consent was obtained from participants approving to join the current trial. Recruitment started on 1-1-2023 and continued for one month. Study was conducted between 1-2-2023 and 1-2-2025.

### Allocation

**Fig. 2:**
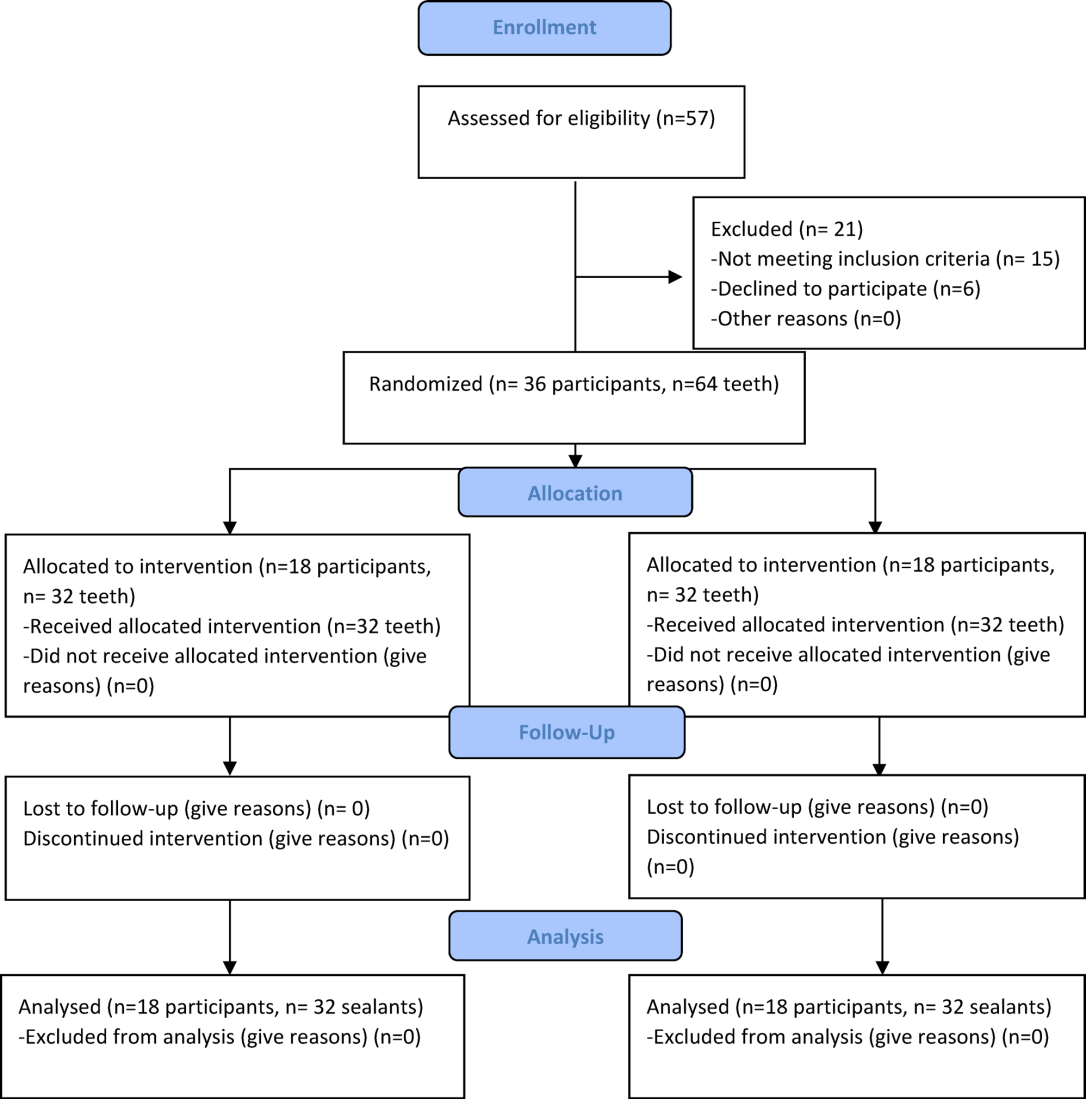
CONSORT flow diagram: enrollment and final analysis overview of the number of participants assessed for eligibility, randomized, and analyzed in the final outcome assessments.

The sequence of participants was generated using simple randomization by generating numbers from 1:36 into two columns (www.random.org). The 36 participants were divided randomly between the intervention groups (n = 18). These 36 participants had 64 teeth with initial carious lesions, that were divided equally between the two interventions (n = 32) fulfilling the calculated sample size. Generated sequence was concealed in opaque sealed envelopes and was only revealed before beginning the restorative procedures. Allocation and enrollment were implemented by a resident who was not engaged in any steps of the present study. The present clinical trial was double blinded to the assessors and participants, differences in the manufacturers’ instructions and color of sealants prohibited blinding of the operator. Flow of participants through each stage of the randomized clinical trial, including the numbers screened for eligibility, randomized, allocated to interventions, followed up, and included in the final analysis is shown in Fig. 2.

### Oral hygiene instructions

Prior to interventions basic prophylaxis was implemented according to the clinical guidelines of CAMBRA^21,23^. Participants were advised to brush their teeth twice daily using toothpaste containing 1450 ppm fluoride, rinse using 0.12% chlorohexidine mouthwash for 1 min once per day for one week one hour apart from fluoride toothbrushing and limit snacks between meals.

### Interventions

In the present study, the intervention group received Biocoat® Sealant (Premier®, Plymouth Meeting, PA, USA; LOT 1511514), a 56% filled resin formula incorporating rechargeable SmartCap microcapsules containing ionic solutions of fluoride, calcium, and phosphate, it was preceded with Premier® Etch (LOT 3001422), a 37% phosphoric acid gel with fumed silica. The control group was treated with Clinpro™ Sealant (3M ESPE, St. Paul, MN, USA; LOT 1507275), composed of 2,2-bis[4-(2-hydroxy-3-methacryloxypropoxy) phenyl]propane, tri(ethylene glycol) dimethacrylate, a light-cured initiator system based on camphorquinone, tertiary amine, iodonium salt, silane-treated fumed silica (6 wt%), titanium dioxide, a patented organic fluoride salt, and rose bengal dye, it was preceded with Scotchbond™ Universal Etchant (LOT 494871), which contained 32% phosphoric acid, water, synthetic amorphous silica, polyethylene glycol, and aluminum oxide.

Interventions were implemented by a single operator (A.A.). The teeth to be sealed was anesthetized using Articaine HCL 4% 1:100.000 (Art Pharma Dent Pharmaceuticals, Giza, Egypt) and the operating field was isolated by rubber dam following the multiple isolation technique after selection of suitable clamps (KSK dentech, Tokoyo, Japan) and sheets (Sanctuary Dental Dam, Perak, Malaysia). Occlusal surface was polished using fluoride free polishing paste (Mira-Clin P, Hager & Werken, Duisburg, Germany) and polishing brush (Epic Europe, Novi Sad, Serbia). All materials were applied according to the manufacturers’ instructions.

Biocoat (premier, Plymouth meeting, PA, USA)

Prior to sealant application, pits and fissures were etched using 37% phosphoric acid (Premier Etch, Plymouth Meeting, PA, USA) for 20 s, then the etchant was thoroughly rinsed using water for 20 s. Afterwards the etched surface was gently air dried until it appeared matte frosty white. Biocoat sealant was applied using the dispensing tip into the pits and fissures with stirring motion, then light cured using LED light curing unit (I LED, Guilin Woodpecker Instruments Co., Guilin, Guangxi, China) of an intensity of 1000–2500 mW/cm^2^ for 20 s.

Clinpro (3M ESPE, St. Paul, MN, USA):

Before sealant application, pits and fissures were etched using 32% phosphoric acid (Scotchbond™ Universal Etchant, 3M ESPE, St. Paul, MN, USA) for 20 s, thoroughly rinsed using water for 20 s, then etched surface was gently dried using air from the dental unit, until it appeared matte frosty white after dryness. The pink Clinpro sealant was applied using the dispensing tip following stirring motion, then light cured using LED light curing unit for 10 s, till its color was transformed from pink to opaque off-white color.

### Outcome assessment

Retention was assessed using Simonsen’s criteria^24^, which categorized sealant retention into complete retention, partial or total loss. The **“complete retention”** indicated that some fissures were revealed at the edges due to wear but no ledges were observed; **“partial loss”** referred to exposure of a portion of the pit or fissure following wear or material loss; and **“total loss”** indicated that no remnants or trace of the sealant could be detected. The assessment of retention was conducted using a dental explorer and mirror, teeth were air-dried and observed under standardized illumination and chair position. Inhibition of initial carious lesion progression was assessed using light fluorescent camera (VistaProof, Dürr Dental, Bietigheim-Bissingen, Germany)^22^, which categorized inhibition of caries progression into yes or no. **“Yes”** represented no caries progression around pits and fissures or at pre-sealed fissures even if the sealant was partially or completely lost (VistaProof reading ≤ 0.9), while **“No”** indicated caries progression in these areas when the sealant was partially or completely lost (VistaProof reading > 0.9). A spacer was utilized for each tooth to ensure that the distance between the camera tip and the tooth was standardized.

Prior to the main study, the two assessors (O.S. & H.H.) underwent calibration and training process to ensure inter-examiner reliability. Both assessors held PhD degree and possessed over 15 years of clinical and research experience in the dental field. The calibration involved the assessment of 10 pre-sealed teeth, where each assessor independently evaluated the sealed teeth using Simonsen’s criteria and VistaProof. Any discrepancies in their assessments were discussed and resolved to achieve consensus, thereby standardizing their evaluation approach for the subsequent study, calibration and training revealed almost perfect inter-observer agreement (Kappa coefficient = 0.91) between assessors. Retention and inhibition of initial carious lesion progression of both sealants was assessed after 12 and 24 months by the two blinded, calibrated assessors according to the above-mentioned criteria, if there was disagreement in any assessment, a third assessor was invited to resolve the conflict. Sealants with total loss and caries progression were considered failure.

### Statistical analysis

Statistical analysis was performed using Medcalc 22 for windows (MedCalc Software Ltd, Ostend, Belgium). Retention and inhibition of initial carious lesion progression data were described as frequency (n) and percentage (%). Intergroup comparison between sealants within each follow-up period was performed using the Chi square test with statistical significance set at (*p* ≤ 0.05), while intragroup comparison within each sealant to assess change in clinical performance through time was performed by the Cochran’s Q test followed by multiple comparisons with statistical significance set at (*p* ≤ 0.016) after Bonferroni correction. The clinical effect size was determined using relative risk (RR). Survival analysis was performed using Kaplan–meier and Log-rank test. Spearman’s correlation was used to correlate sealant retention to inhibition of initial carious lesion progression. Logistic regression was used to assess the relationship between age, gender, teeth, sealant material and success of fissure sealants.

## Results

### Demographic data

The present trial was conducted on 36 participants (n = 18) per group, with 64 non-cavitated occlusal carious lesions (n = 32) per group. After 24 months all sealants were assessed, with 100% recall rate. Demographic data of participants and teeth is shown in Table 1, there was no statistically significant difference between both groups regarding age, gender and teeth distribution (*p* > 0.05).

**Table 1:** Demographic data of participants and teeth recruited in the current study.

Gender	Biocoat	Clinpro	Row total (RT)
Male	7	9	16 (44.4%)
Female	11	9	20 (55.6%)
**Column total (CT)**	18 (50.0%)	18 (50.0%)	36
**P value**	*p* = 0.5083		

Teeth	Biocoat	Clinpro	Row total (RT)
Maxillary 1st molar	5	11	16 (25%)
Maxillary 2nd molar	5	9	14 (21.8%)
Maxillary 3rd molar	2	2	4 (6.25%)
Mandibular 1st molar	11	8	19 (29.68%)
Mandibular 2nd molar	9	2	11 (17.27%)
**Column total (CT)**	32 (50.0%)	32 (50.0%)	64
**P value**	*p* = 0.0805		

Age	Mean	SD	Average
Biocoat	26.6	4.9	25.4 ± 4.7
Clinpro	25.1	4.6	
P value	*p* = 0.730		

### Retention

Comparisons of sealant retention between Biocoat and Clinpro sealants have shown no statistically significant difference at baseline and after 12 months (*p* = 1.0000 and 0.5251) respectively, while after 24 months there was statistically significant difference (*p* = 0.0170). Comparison within Biocoat and Clinpro sealants has shown statistically significant change in sealant retention with time (*p* = 0.0004 and *p* < 0.0001) respectively. There was 93% less risk for total loss of Biocoat sealant when compared to Clinpro sealant after 24 months (RR = 0.07 (95% CI 0.004513 to 1.311; *p* = 0.0763)). (Table 2).

**Table 2:** Frequency (n) and percentage (%) for retention and inhibition of initial carious lesion progression scores for the intergroup comparison between materials within each follow-up and intragroup comparison within each material between different follow-up periods.

Retention	Biocoat(n = 32)			Clinpro(n = 32)			P value
	Complete retention	Partial loss	Total loss	Complete retention	Partial loss	Total loss	
Baseline	32(100%)	0(0%)	0(0%)	32(100%)	0(0%)	0(0%)	*p* = 1.0000
12 months	27(84.3%)	5(15.7%)	0(0%)	25(78.1%)	7(21.9%)	0(0%)	*p* = 0.5251
24 months	20(62.5%)	12(37.5%)	0(0%)	12(37.5%)	14(43.75%)	6(18.75%)	*p* = 0.0170*
**P value**	*p* = 0.0004*			*p* < 0.0001*			

Caries Inhibition	Yes (≤ 0.9)		No (> 0.9)	Yes (≤ 0.9)		No (> 0.9)	P value
Baseline	0(0%)		32(100%)	0(0%)		32(100%)	*p* = 1.0000
12 months	32(100%)		0(0%)	30(93.75%)		2(6.25%)	*p* = 0.1540
24 months	30(93.75%)		2(6.25%)	23(71.8%)		9(28.2%)	*p* = 0.0214*
**P value**	*p* < 0.0001*			*p* < 0.0001*			

### Inhibition of initial carious lesion progression

Comparisons of inhibition of initial carious lesion progression between Biocoat and Clinpro sealants have shown no statistically significant difference at baseline and after 12 months (*p* = 1.0000 and *p* = 0.1540) respectively, while after 24 months there was statistically significant difference (*p* = 0.0214). Comparison within Biocoat and Clinpro sealants has shown statistically significant change in inhibition of initial carious lesion progression with time (*p* < 0.0001). There was 78% less risk for caries progression using Biocoat sealant when compared to Clinpro sealant after 24 months (RR = 0.22(95% CI 0.05204 to 0.949; *p* = 0.0423)). (Table 2).

### Survival analysis

**Fig. 3:**
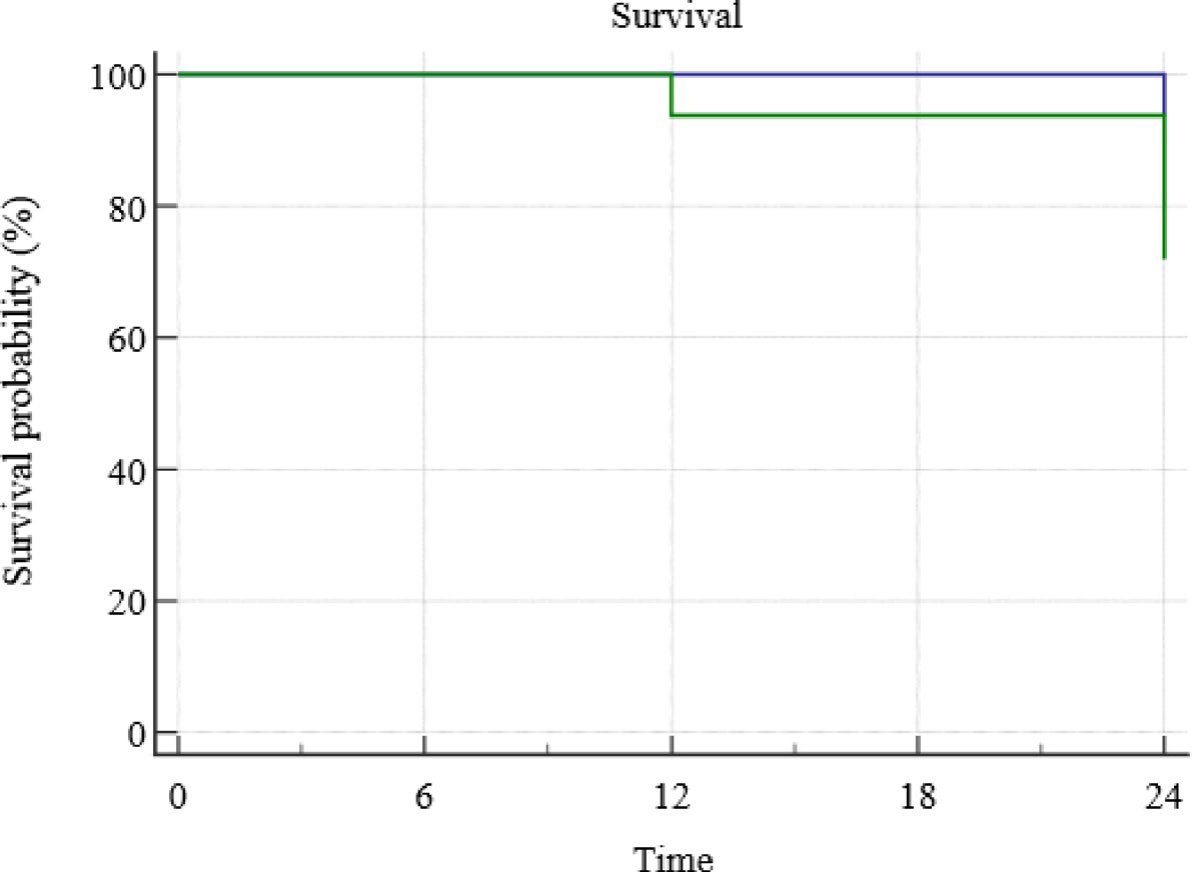
Survival analysis of Biocoat and Clinpro sealants after 24 months in non-cavitated occlusal caries.

Survival analysis after 24 months has shown that two sealants in Bioacoat sealant group and 11 sealants in Clinpro sealant group failed due to total loss of retention and caries progression, showing statistically significant difference between both sealants (*p* = 0.0197). (Fig. 3) Completely retained sealants with no evidence of caries progression were considered successful. Biocoat has shown 93.75% success rate, while Clinpro sealant has shown 65.62% success rate after 24 months. There was 82% less risk of failure of Biocoat sealant when compared to Clinpro sealant after 24 months (RR = 0.18 (95%CI 0.04374 to 0.7558, *p* = 0.0190)), showing a statistically significant difference between both sealants after 24 months.

### Correlation between sealant retention and inhibition of initial carious lesion progression

Within Biocoat sealant, there was moderate positive correlation between sealant retention and inhibition of initial carious lesion progression (rho = 0.3, *p* = 0.1092), while within Clinpro sealant, there was strong positive correlation (rho = 0.4, *p* = 0.0319).

### Logistic regression

Sealant material and teeth showed statistically significant relationship with success of sealants. There were 89% lower odds of failure of Biocoat when compared to Clinpro sealant (OR = 0.11 (95%CI 0.01 to 0.75, *p* = 0.0245)), also maxillary teeth showed 10.8 more times the odds of failure when compared to mandibular teeth (OR = 10.84 (95%CI 1.61 to 72.63, *p* = 0.014)). (Table 3).

**Table 3:** Logistic regression showing relationship between age, gender, teeth, sealant material and success of fissure sealants.

Variable	Coefficient	Std. Error	Odds ratio	95%CI	P value
Constant	2.32	4.23			*p* = 0.5831
Age	0.08	0.10	1.08	0.89 to 1.31	*p* = 0.4239
Gender	-1.51	0.87	0.22	0.04 to 1.20	*p* = 0.0807
Material	-2.21	0.98	0.11	0.01 to 0.75	*p* = 0.0245*
Teeth (Jaw)	2.38	0.97	10.84	1.61 to 72.63	*p* = 0.014*

## Discussion

Interestingly, dental restoration techniques have significantly evolved. Previously, treatments were heavily focused on operative interventions. However, there is now a global shift towards minimally invasive approaches, which are supported by extensive scientific research. This conservative approach focuses on the non-surgical management of early non-cavitated carious lesions and the implementation of effective minimally invasive treatments for dentinal caries^23^. Modern caries management prioritizes early diagnosis and prevention of non-cavitated lesions. However, some dentists continue to favor surgical interventions, which can initiate a cycle of repeated restorations, increasing the risk of pulp damage and affecting adjacent teeth. This cycle may compromise the longevity of both the tooth and the restoration. Embracing non-invasive treatments is expected to reduce secondary caries, prevent early restoration failures, lower the risk of tooth fractures due to extensive restorations, and preserve pulp vitality for a longer duration^25,26^. The concept of sealing carious lesions is not new, previous 10 years clinical trials^27,28^ found that sealing cavitated carious lesions with resin composite was capable of arresting caries progression.

After 24 months Biocoat has shown better retention rate and inhibition of initial carious lesion progression when compared to Clinpro sealant, therefore the null hypothesis was rejected. Regarding fluoride releasing sealants, previous results by Borges et al.^26^, has shown 11.5% caries progression after 12 months, while there were 3.8% total loss of sealants after 24 months. Another study by Bakhshandeh et al.^17^, reported 14% complete loss of fluoride releasing sealants after 2–3 years, however they reported 10% caries progression. Moreover, a previous research by Alves et al.^29^, reported 76% success rate of fluoride releasing sealants after 3–4 years, which is comparable to the present results with a success rate of 65.62%. This difference in the caries progression rate between previous studies and the present clinical trial may be attributed to the sensitivity of the fluorescent camera for detection of presence and progression of dental caries. Previous research^22^ revealed high sensitivity of VistaProof fluorescent camera for detection of pit and fissure enamel and dentin caries (92.3% and 74.1%) respectively. For monitoring caries progression, VistaProof has shown high sensitivity and specificity after cariogenic challenge of 87% and 99% respectively^30^.

According to a previous study by Berdouses et al.^31^, sealing initial occlusal caries using fluoride releasing fissure sealant showed double the probability to fail when compared to sound surfaces. Initial carious lesions treated with fluoride releasing sealants were 100% more likely to require subsequent restoration and have a 69% higher probability of sealant failure compared to sound surfaces. This suggested that fluoride releasing sealants applied to non cavitated occlusal carious lesions are less protective than anticipated.

The increased likelihood of sealant failure on occlusal surfaces with initial carious lesions can be attributed to two primary factors. The first factor is the increased cariogenic environment in high-risk patients. High-risk patients exhibit elevated bacterial loads, including *Streptococcus mutans*, which create a highly cariogenic environment. This bacterial activity often overwhelms the protective effects of sealants^32^. These bacteria, in combination with salivary enzymes such as esterases, degrade resin composites and adhesives through hydrolytic activity. Esterases compromise the resin-dentin interface by degrading the polymer matrix, leading to marginal demineralization and allowing bacteria to infiltrate and undermine the sealant. This process significantly shortens the lifespan of resin-based restorations, particularly in high-risk individuals^33,34^.

The second contributing factor is the altered enamel characteristics in early carious lesions. The condition of the enamel plays a crucial role in sealant retention. Effective sealing requires strong micromechanical bonding, achieved by forming resin tags in a properly conditioned enamel surface. However, this ideal scenario applies primarily to sound enamel. In early carious lesions, fluoride incorporation and organic material deposition within enamel micro spaces during lesion formation increase acid resistance but reduce resin penetration and bonding strength. These altered enamel surfaces have higher porosity, increased protein content, and reduced calcium, phosphorus, and carbonate levels. This compromised structure has been shown to result in significantly greater microleakage compared to sound enamel, especially after thermal and mechanical stresses. Microleakage allows saliva and bacteria to infiltrate the tooth-sealant interface, undermining the sealant’s integrity and promoting failure^35,36^.

Previous literature claimed that 85% of sealants are completely intact after 1 year^17^. Biocoat sealant attained this retention rate by having 84.3% completely sealed fissures, while Clinpro sealant failed to achieve this rate by having only 78.1% completely sealed fissures. If a fissure sealant is partially or completely lost, it may create a stagnation area that promotes the initiation and progression of caries^17^. Therefore, materials with caries-inhibitory and remineralizing properties are crucial. The incorporation of fluoride in resin-based sealants has been employed for caries prevention, as fluoride release helps inhibit enamel demineralization by forming a fluoride-rich layer that enhances resistance to caries, even in the absence of the sealant^37^. When a sealant is partially lost, deep fissures remain unprotected, facilitating biofilm accumulation and subsequent caries development. However, the caries-preventive effect may persist if residual sealant remains within the depths of pits and fissures^38^.

In the present study only 2 sealants (6.25%) showed caries progression in Biocoat group, while 9 sealants (28.2%) showed caries progression in Clinpro group. Therefore, fluoride was not that effective in inhibiting caries progression. The glass filler particles in fluoride resin-based materials serve as the fluoride source, resulting in a gradual, diffusive release. Clinpro sealant’s fluoride release pattern starts out high and then gradually decreases with time until it hits a plateau, this may be the cause of the poor inhibition of initial carious lesion progression effect of Clinpro fissure sealant^39^.

The unique SmartCap™ technology from Premier® could be responsible for the excellent performance of Biocoat sealant. In this technology, semi-porous resin microcapsules with a polyurethane-based shell that serves as a semi-permeable membrane are used. The fluoride, calcium, and phosphate ionic solutions in the microcapsules can diffuse in and out of the sealant in response to concentration gradients, improving the adjacent tooth structure’s ability to absorb the ions. In addition to strengthening enamel and preventing demineralization during acid attacks, this condition of ion supersaturation also closes margins against microleakage. Additionally, aqueous solutions containing calcium, phosphate, and fluoride can enter the microcapsules and refill their contents, since the microcapsule shell permits two-way permeability^40,41^.

The literature lacks sufficient evidence-based information about the recently introduced Biocoat sealant. Only two clinical trials are available. Both trials were assessing Biocoat in sealing sound pits and fissure. Abdelsalam et al.^7^, found no statistically significant difference in retention rates between Biocoat and fluoride releasing sealant after 12 months, however Biocoat sealant provided better inhibition of initial carious lesion progression. Moreover Ergün and Nahir^42^, found no difference between Biocoat and fluoride releasing sealant after 6 months in retention and marginal adaptation. The similar retention rate between Biocoat and fluoride releasing sealant in these studies may be attributed to using them in sound pits and fissures. In the present trial carious pits and fissures were sealed, as mentioned earlier, the condition of enamel affects the sealant retention, this may be the cause of the different retention rate.

After 24 months, there were moderate to strong positive correlations between sealant retention and inhibition of initial carious lesion progression. This means that as the retention increases the inhibition of initial caries progression increases. The arrest of caries is highly dependent on the retention of sealants. The inhibition of nutrient supply to infected dentin due to sealant retention may account for the absence of caries progression. However, if the sealant bond is compromised, the restoration of nutrient flow can reactivate bacterial growth, leading to further caries development^28,43^.

Logistic regression revealed statistically significant relationship between teeth and sealant success. Maxillary teeth experienced higher sealant failure rates, accounting for 11 (84%) of the 13 total failed teeth. Mandibular teeth exhibited superior retention, likely due to direct visibility, gravity assisting sealant flow, and well defined pits and fissures^44^.

Limitations of the present clinical trial include small sample size, which may affect the generalizability of the findings and 24 months observation period which is considered intermediate. Another limitation of the current study is the retrospective nature of the trial registration. Although the protocol and analysis plan were finalized prior to patient enrollment, the delayed registration is a departure from best-practice standards. We acknowledge that this administrative delay introduces a theoretical risk of reporting bias. To our knowledge, the present clinical trial is pioneer in assessing Biocoat sealant with the innovative SmartCap™ technology developed by Premier® in sealing non-cavitated occlusal caries. Larger sample size and at least 3-year observation period are recommended to confirm the current results.

## Conclusions

Biocoat sealant showed better retention rate and inhibition of initial carious lesion progression after 24 months when compared to fluoride releasing sealant in non-cavitated occlusal caries in adults. The AAPD advised strong recommendation of moderate quality of evidence on sealing initial non-cavitated occlusal caries in children and adolescents. The panel suggested extending this recommendation to adults. The present study supports this suggestion, as sealing of initial non-cavitated occlusal carious lesion is a conservative and successful approach and should be implemented in routine dental practice in adults with recent history of dental caries.

## Data Availability

The datasets used and/or analysed during the current study available from the corresponding author on reasonable request.

## Abbreviations

ADA CSA: American dental association council on scientific affairs
AAPD: American academy of pediatric dentistry
CAMBRA: Caries management by risk assessment
CONSORT: Consolidated standards of reporting trials
JSCD: Japanese society of conservative dentistry
LED: Light emitting diode
OR: Odds ratio
RR: Relative risk
